# Advancements in Human Breast Cancer Targeted Therapy and Immunotherapy

**DOI:** 10.7150/jca.64205

**Published:** 2021-10-11

**Authors:** Mayassa J. Bou-Dargham, Sophia Draughon, Vance Cantrell, Zahraa I. Khamis, Qing-Xiang Amy Sang

**Affiliations:** 1 Department of Chemistry and Biochemistry, Florida State University, Tallahassee, Florida, United States of America.; 2 Department of Chemistry and Biochemistry, Faculty of Sciences-I, Lebanese University, Beirut, Lebanon.; 3 Institute of Molecular Biophysics, Florida State University, Tallahassee, Florida, United States of America.

**Keywords:** Human breast cancer, targeted therapy, immunotherapy

## Abstract

Human breast cancer treatment regimens have evolved greatly due to the significant advances in understanding the molecular mechanisms and pathways of the common subtypes of breast cancer. In this review, we discuss recent progress in breast cancer targeted therapy and immunotherapy as well as ongoing clinical trials. We also highlight the potential of combination therapies and personalized approaches to improve clinical outcomes. Targeted therapies have surpassed the hormone receptors and the human epidermal growth factor receptor 2 (HER2) to include many other molecules in targetable pathways such as the epidermal growth factor receptor (EGFR), poly (adenosine diphosphate-ribose) polymerase (PARP), and cyclin-dependent kinase 4/6 (CDK4/6). However, resistance to targeted therapy persists, underpinning the need for more efficacious therapies. Immunotherapy is considered a milestone in breast cancer treatments, including the engineered immune cells (CAR-T cell therapy) to better target the tumor cells, vaccines to stimulate the patient's immune system against tumor antigens, and checkpoint inhibitors (PD-1, PD-L1, and CTLA4) to block molecules that mediate immune inhibition. Targeted therapies and immunotherapy tested in breast cancer clinical trials are discussed here, with special emphasis on combinatorial approaches which are believed to maximize treatment efficacy and enhance patient survival.

## Introduction

Despite the recent medical advancements, breast cancer remains the most common malignancy and the second deadly cancer among women according to the Centers for Disease Control and Prevention (CDC 2016) 1. In 2021, it is estimated that breast cancer will affect 2811,550 women and cause the death of 43,600 women in the United States 2. Like other cancers, breast cancer shows inter- and intra-tumor heterogeneity. Inter-tumor heterogeneity occurs among various individuals and is elucidated by the TNM (tumor-nodes-metastasis) staging system, the histopathologic classification, and the tumor grade 3. The breast cancer clinical staging system runs from I-IV and is based on tumor size, lymph node involvement, and distant metastases 4. The morphologic heterogeneity of breast carcinomas is evaluated based on histological type and histological grade that take into consideration the growth pattern and the degree of differentiation of the tumor, respectively 5. The major histologic types of breast cancer involve invasive ductal carcinoma (50%-75%) and invasive lobular carcinoma (5%-15%) with the remainder showing mixed or other special histology 4. The other morphologic heterogeneity, the tumor grade, depends on three architectural features, namely the degree of tubule or gland formation, nuclear pleomorphism, and mitotic rate 5. On the other hand, breast cancer intra-tumor heterogeneity is observed at the morphologic, genomic, transcriptomic, and proteomic levels, and is due to the presence of distinct cellular phenotypes within the same individual tumor. The aforementioned heterogeneities in breast carcinomas have a dire impact on diagnosis, prognosis, and treatment of the disease.

While clinical and morphologic heterogeneities are important 6, expression heterogeneity is one of the main parameters used to classify breast tumors. Expression heterogeneity is mostly observed in the differential expression of the hormone receptors (HR), estrogen and progesterone, and the human epidermal growth factor receptor 2 (HER2/ERBB2). The major subtypes of human breast cancer are luminal A, luminal B, HER2-enriched (HER2^+^), and triple-negative breast cancers (TNBC). Luminal A and B are the hormone receptor positive (HR^+^) subtypes characterized by the expression of the estrogen receptor (ER^+^) and progesterone receptor (PR^+^) and generally respond to hormone therapy 7. Unlike the luminal A subtype, luminal B breast cancers show lower expression of hormone receptors and higher expression of proliferation markers and HER2. In general, both luminal A and luminal B may be associated with a good prognosis and long-term survival due to many available treatments 8. However, the HER2-enriched subtype, characterized by HER2 overexpression, is responsive to anti-HER2 antibodies but is associated with a poor clinical outcome. The efficacy, safety, dosage, and mode of administration of HER2-targeted drugs are still under investigation in clinical settings 9. The triple-negative breast cancer (TNBC) lacks the expression of the hormone receptors and the amplification of HER2, and commonly overexpresses the epidermal growth factor receptor (EGFR). TNBC is further stratified into basal-like, normal breast-like, and claudin-low molecular subtypes 10,11. While hormone therapies have shown good results for hormone receptor-positive patients during early stages, the treatment of late-stage and triple-negative breast cancers remains challenging.

Treatment of breast cancer has evolved greatly in recent years due to significant advances in understanding the molecular mechanisms and pathways of the most common subtypes of breast cancer. In this review, we discuss recent progress in targeted therapy and immunotherapy as well as ongoing clinical trials in breast cancer. We also highlight the outcomes of combined therapies and suggest the potential application of combination therapy and personalized approaches to improve clinical outcomes.

## Evolution of Breast Cancer Treatments

To determine the optimal therapeutic protocol, breast cancer stage and subtype must be identified. In the United States, 62% of patients suffer from localized breast cancer upon diagnosis, while 31% have tumors spread to sentinel lymph nodes and 6% to distant sites. Treatment strategies for patients with nonmetastatic disease aim to eradicate the tumor from the breast tissue and adjacent lymph nodes, and to prevent relapse 4. Local treatment of nonmetastatic breast cancer involves surgical resection (lumpectomy or mastectomy) with regional lymph node biopsy or full dissection that might be followed by adjuvant radiotherapy 12. For early-stage (I or II) tumors, most patients undergo lumpectomy plus adjuvant radiotherapy (49%); whereas 34% of patients choose mastectomy alone or combined with chemotherapy/radiotherapy. The most common treatment among patients diagnosed with stage III breast cancer is mastectomy with adjuvant chemotherapy/radiotherapy (68%). As for metastatic breast cancer (IV), the treatment options tend to be palliative rather than curative with 56% of women receiving radiation therapy/chemotherapy alone 13.

Breast cancer subtype is relied upon to determine the optimal treatment for all stages of the disease and dictates the administered systemic therapy 12. The standard systemic therapy for metastatic and nonmetastatic breast cancer includes endocrine therapy for HR^+^ tumors, HER2-targeted agents for HER2^+^ tumors, and chemotherapy alone for TNBC. Despite its risks, neoadjuvant and adjuvant chemotherapy continues to be an important adjunct for the systemic treatment of patients with HR^+^ and ERBB2^+^ breast cancers 4. Targeted therapy against hormone receptors and HER2 often leads to resistance in clinical settings. Thus, alternative molecular agents in targetable pathways are urgently needed to overcome resistance and ameliorate the clinical outcome. In addition, other treatment modalities are being investigated to obviate the toxicities associated with various therapies. One such approach is immunotherapy that activates the patient's immune system by arming it with the weapons necessary to destroy the tumor cells.

## Targeted Therapies

### EGFR Targeted Therapies

**Figure 1:**
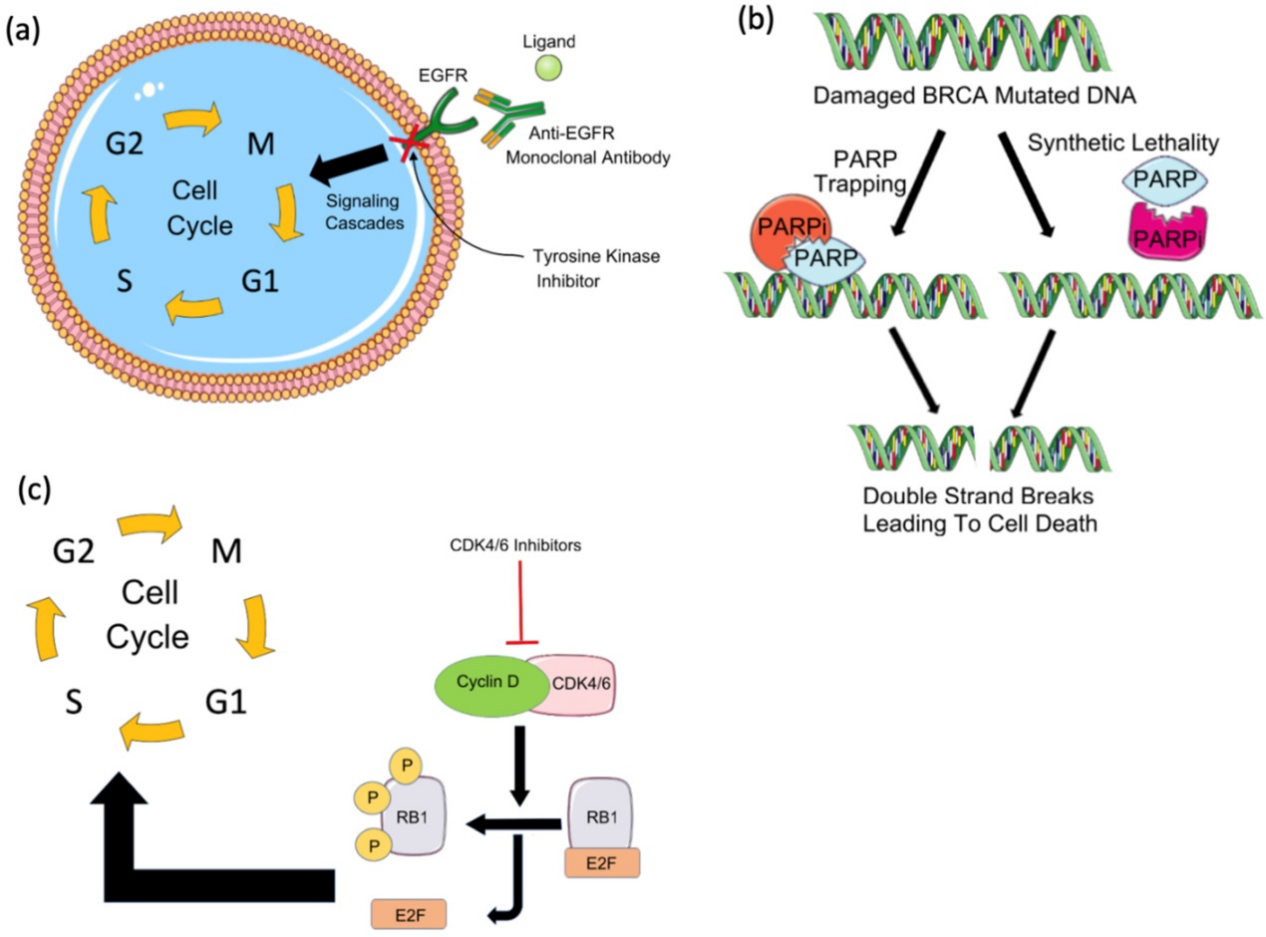
** Targeted therapies in breast cancer. (a)** Tyrosine kinase inhibitors and EGFR monoclonal antibodies against EGFR block the downstream signaling cascade that inhibits cell cycle progression. **(b)** PARP inhibitors in BRCA mutated patients result in cancer cell death by synthetic lethality due to BRCA mutation or by trapping PARP on the DNA and interfering with its catalytic activity by blocking its auto-PARylation. (c) Cyclin-dependent kinase (CDK) inhibitors such as CDK4/6 inhibitors block the activation of cyclin D and downstream cell cycle progression by preventing the release of Rb and E2F.

The majority of TNBC tumors have increased EGFR expression suggesting this receptor as a potential therapeutic target to treat TNBC. Currently, two types of EGFR-targeted therapies - including small-molecule tyrosine kinase inhibitors and monoclonal antibodies, are being examined in ongoing clinical trials for the treatment of TNBC (Figure 1A) 10. Gefitinib, an EGFR tyrosine kinase inhibitor, was tested for its ability to treat breast cancer 14. The efficacy of gefitinib alone or in combination with neoadjuvant epirubicin and cyclophosphamide (EC) was evaluated in estrogen receptor-negative invasive breast cancer patients. While patients receiving EC with or without gefitinib showed no significant difference in the overall survival, TNBC patients showed a significantly higher pathologic complete response compared to non-TNBC patients independent of the treatment 14.

Another trial assessed the effectiveness of cisplatin (an alkylating agent interfering with DNA replication) alone or in combination with cetuximab (anti-EGFR agent) in metastatic TNBC. The objective response rate was higher in the patient group that received cetuximab in combination with cisplatin (20%) compared to the group receiving cisplatin alone (10%), but the results were statistically insignificant 11.

### PARP Inhibitors

Poly(ADP-ribose) polymerase (PARP) inhibitors (PARPi) are drugs that mainly target breast cancer patients with germline BRCA1 or BRCA2 mutations 15. Of the PARP enzyme family, PARP-1 plays a key role in signaling DNA damage and mediating base excision repair. Through polymerization of ADP-ribose (PARylation), PARP-1 repairs single-strand breaks (SSBs) through the base excision repair pathway. If PARylation is blocked, SSBs will accumulate and transform into double-stranded breaks (DSBs), which require homologous recombination (HR) for repair. The failure of HR to repair DSBs results in genomic instability and subsequent cell death. Cells deficient in HR, like those with BRCA1 or BRCA2 mutations, are sensitive to PARP inhibitors and usually die by synthetic lethality or PARP trapping. Synthetic lethality depends on the deadly effect of the mutation of two alleles on a cell or organism 15,16. While in PARP trapping, PARPi traps the PARP-1 enzyme on DNA preventing its autoPARylation and its release from the site of damage; thus, interfering with the catalytic cycle of PARP1 and causing irreparable DNA damage (Figure 1B) 17.

The FDA approved the PARP inhibiting drugs talazoparib and olaparib for use in breast cancer. FDA approval of talazoparib was based on the EMBRACA trial. This trial investigated the effects of talazoparib compared to standard therapy in patients with BRCA-mutation breast cancer 18. EMBRACA results showed that the patients who received talazoparib had an overall response rate of 62.6% compared to 27.2% in patients receiving chemotherapy. The initial results also showed that the talazoparib group had a significantly higher progression-free survival of 8.6 months compared to 5.6 months in the standard therapy group 19.

Olaparib's FDA approval was based on the OlympiAD trial which compared chemotherapy to olaparib monotherapy in patients with HER2^-^, BRCA-mutated metastatic breast cancer 19,20. OlympiAD found that the median progression-free survival and the overall survival were both higher in the patients who received olaparib than in the patients who received chemotherapy. The response rate was 60% in the olaparib group compared to only 28.8% in the chemotherapy group 19. Collectively, EMBRACA and OlympiAD trials demonstrate the effectiveness of PARP inhibitors as a therapy for breast cancers with BRCA mutations.

### CDK4/6 Inhibitors

Cyclin-dependent kinase (CDK)4/6 plays a key role in cellular proliferation. By phosphorylating retinoblastoma (Rb), CDK4/6 induces the release of the transcription factor E2F which activates the expression of genes needed for DNA replication and thereby, promoting the transition of the cell cycle from the G1 to the S phase (Figure 1C) 21. The CDK4/6-Rb interaction increases cancer cells' survival and CDK4/6 is known to play a role in breast cancer pathogenesis and tumorigenesis 22. CDK4 was found to be amplified in 14% of luminal A and 25% of luminal B breast cancer patients. Cyclin-D was found to be amplified in 29% of luminal A and 58% of luminal B breast cancer patients 21.

CDK4/6 inhibitors have had a great deal of success in treating hormone receptor-positive (HR^+^) and HER2^-^ advanced breast cancer patients. Three CDK4/6 inhibitors were recently approved for the treatment of HR^+^/HER2^-^ breast cancer patients: palbociclib, ribociclib, and abemaciclib 23. Palbociclib was granted accelerated approval in 2015 with the aromatase inhibitor letrozole for the treatment of ER^+^/HER2^-^ advanced breast cancer as an initial endocrine-based therapy in postmenopausal women. In 2017, the FDA granted regular approval for palbociclib in combination with another anti-estrogen drug, fulvestrant, for the treatment of HR^+^/HER2^-^ advanced or metastatic breast cancer in women who have developed disease progression following an endocrine therapy 23. The approval of palbociclib was based on a series of trials known as the Paloma trials, conducted on HR^+^/HER2^-^ breast cancer patients. Paloma I, II, and III demonstrated the ability of palbociclib to improve progression-free survival for breast cancer patients 23,24. In April 2019, the FDA extended the approval of palbociclib to include men 25.

The results of the MONALEESA trials granted ribociclib FDA approval as a first-line treatment for HR^+^/HER2^-^ advanced breast cancer in postmenopausal women 23. In 2018, the FDA approved the combination of ribociclib with an aromatase inhibitor as an initial endocrine-based therapy for pre- or perimenopausal women with HR^+^/HER2^-^ advanced or metastatic breast cancer. A series of clinical trials known as the MONARCH trials in HR^+^/HER2^-^ patients led to the approval of another CDK4/6 inhibitor known as abemaciclib. Abemaciclib was first approved in 2017 for either second or later-line therapy with fulvestrant. It was also approved for either third or later-line therapy for both men and women as a single agent. In 2018, abemaciclib was later approved in combination with an aromatase inhibitor as initial endocrine-based therapy for postmenopausal women with HR^+^/HER2^-^ cancer 23. Novel combinations with CDK4/6 inhibitors and the use of CDK4/6 inhibitors beyond HR^+^/HER2^-^ breast cancer are both areas of current research (Supplementary file 1) 23.

### Anti-HER2 antibodies

As upregulated HER2 expression distinguishes tumor cells from normal breast cells, antibodies against HER2 have emerged as a successful approach for HER2^+^ breast cancer. Although Trastuzumab, a monoclonal antibody targeting HER2, was approved by the FDA for the treatment of metastatic HER2^+^ breast cancer patients in 1998, minimizing its cardiac-related side effects has been the aim of several other trials 26,27.

The standard schedule for trastuzumab monotherapy starts with an initial “loading dose” of 4 mg/kg of body weight followed by a weekly administration of 2 mg/kg. However, the administration of higher doses of trastuzumab may provide a more convenient treatment option if it was not for the major adverse effect of symptomatic cardiac dysfunction observed in about 2% to 4.7% of the patients 9. One phase II trial sought to investigate the efficacy and safety of Trastuzumab administered at a higher dose and longer intervals. In this trial, patients with previously untreated HER2-positive metastatic breast cancer received a median number of five therapy cycles starting with an initial dose of 8 mg/kg and then 6 mg/kg of Trastuzumab intravenously at 3-week intervals until the patients withdrew from the trial or showed disease progression. The study was conducted on 105 HER2^+^ breast cancer patients and showed an overall response rate of 19% with a clinical benefit rate of 33%. Only one patient experienced a cardiac-related adverse event. This patient had a history of obesity and hypertension and was previously on epirubicin therapy which is associated with cardiac toxicity 9,28.

Trastuzumab is tested in combination with chemotherapy in a randomized phase III clinical trial known as the PANTHER. The node-negative breast cancer patients received cyclophosphamide with dose-dependent epirubicin followed by either docetaxel alone or in combination with standard 5-fluorouracil, cyclophosphamide, and epirubicin every three weeks 29. Although long-term cardiac complications were relatively low, the efficacy of the treatment was compromised as the results were not statistically significant 29.

Another anti-HER2 monoclonal antibody, pertuzumab, was examined for its potential to treat breast cancer patients in combination with trastuzumab 30. The CLEOPATRA trial tested the combination of pertuzumab, trastuzumab, and docetaxel against a placebo, trastuzumab, and docetaxel 30. The trial showed that the addition of pertuzumab significantly increased the overall survival rate when compared to the placebo group while maintaining long-term cardiac safety 30. The pertuzumab-receiving group had a median overall survival of 56.5 months whereas the placebo group had 40.8 months 30. Another clinical trial known as NeoSphere showed similar results. Patients treated with pertuzumab, trastuzumab, and docetaxel combination had a significantly improved pathological complete response when compared to patients given only trastuzumab and docetaxel, without significant difference in treatment tolerability 31. In the combination group, 45.8% of patients experienced complete pathological response; whereas the other group showed notably lower pathological complete response 31. Overall, these two studies demonstrate that pertuzumab combined with trastuzumab and docetaxel improves clinical outcomes. More studies targeting HER2 in combination with immunotherapy are discussed in the following sections, and other completed clinical trials are summarized in Supplementary file 1.

## Immunotherapies

### CAR-T Cell Therapy

**Figure 2:**
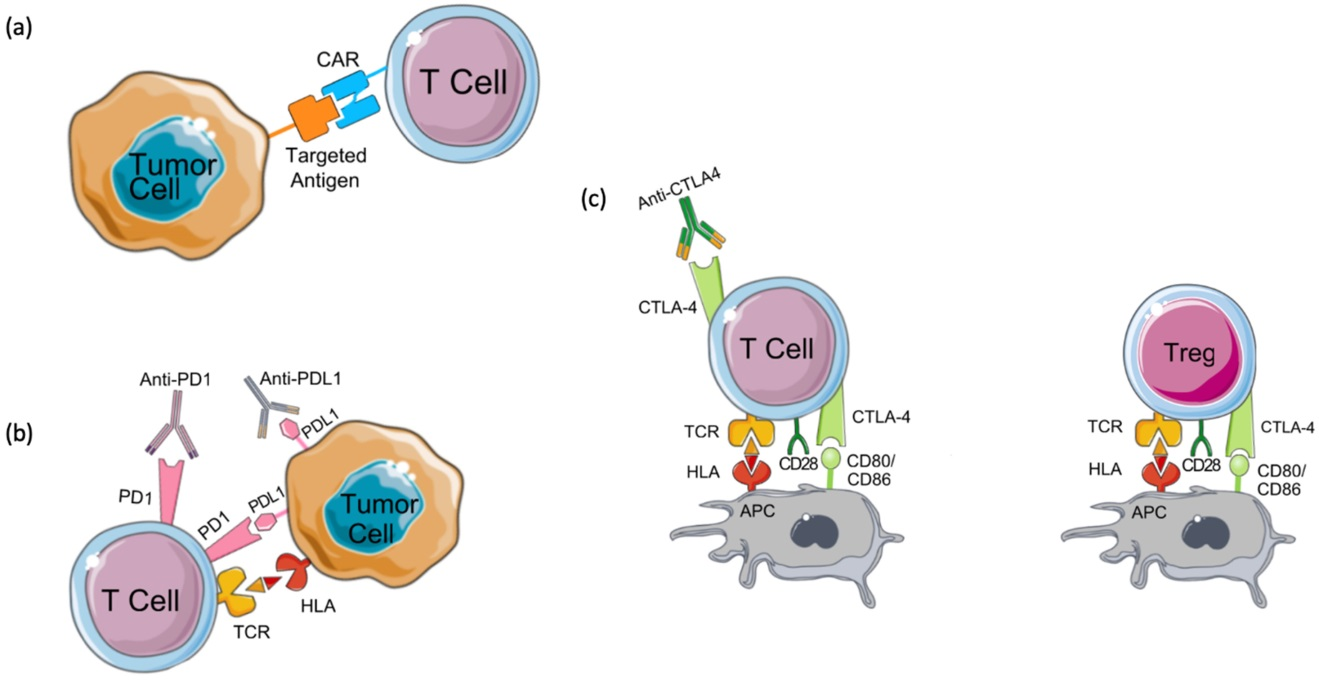
** Mechanisms of action of immunotherapies. (a)** Chimeric antigen receptor (CAR) T cells are engineered to express a chimeric receptor that recognizes an antigen expressed on tumor cells. This recognition results in the activation of cytotoxic T cell immune response targeted against tumor cells. Another class of immunotherapy targets immune checkpoints such as **(b)** programmed cell death-1 (PD-1), its ligand PD-L1, and** (c)** CTLA4. These molecules keep the immune response under control and prevent its over-activation and autoimmunity development. Tumor cells seize this mechanism by expressing PD-L1 which binds to PD-1 expressed on T cells and by inhibiting cytotoxic T cell response. CTLA4, the other immune checkpoint is expressed on regulatory T cells (Tregs) and activated T cells. Tumors induce Treg differentiation and chemotaxis to the tumor microenvironment to inhibit immune activation on one hand, and tolerance and overactivation result in T cell's expression of CTLA4, on the other hand, resulting in the inhibition of co-stimulation and activation of T cells by antigen-presenting cells (APCs) which prime and activate T cells against the tumor antigen. By targeting these immune checkpoints with antibodies that block their functions, tolerance can be overcome, and anti-tumor immune response can be re-activated.

Chimeric antigen receptor-T cell (CAR-T) therapy is a type of T-cell-based adoptive immunotherapy designed to enhance anti-tumor T-cell activity 32. CAR-T cell therapy relies on modifying T cell receptors (TCR) to express chimeric antigen receptors (CARs) that target a specific tumor antigen (Figure 2A) 32,33. CARs are composed of four domains: extracellular, intracellular, transmembrane, and spacer. The extracellular domain is most often built from a single-chain variable fragment (ScFV) of a specific antibody, directed against a target antigen 34. While the transmembrane domain holds the CAR into the cell membrane, the intracellular domain transduces the signals into the cell. Several efforts have been made to enhance the activation and the specificity of CAR-T cells which have resulted in 3 generations of CARs classified depending on the number of costimulatory domains 35,36. Although a perfect CAR structure does not exist yet, the challenge remains in identifying a tumor-associated antigen that allows for a minimal recognition of normal cells' antigens 34,37. Several CAR-T cells have been engineered to target different breast cancer antigens, however, they are still at an early stage and require further validation in clinical trials.

#### AXL-targeted CAR-T

A recent study investigated the expression of AXL, a receptor tyrosine kinase, in breast tumors and cell lines, and the viability of an AXL-targeted CAR-T cell treatment 37. This study tested patient-derived breast cancer tumors for AXL expression and found that patients with TNBC had the highest expression of AXL. AXL-CAR-T cells were co-cultured with the AXL-positive breast cancer cell lines (MDA-MB-231, 786-0, 769-P, Panc1, and MIAPaca2) and the AXL-negative breast cancer cell line, MCF-7. The co-culture resulted in significant cytotoxicity in the AXL-positive cell lines only 37. The *in-vivo* mouse model xenografted with the cell line MDA-MB-231 and treated with AXL-CAR-T cell therapy had the lowest tumor volume compared to the other groups treated with either non-transduced T cells or PBS 37. Although these results point out the promising effects of AXL-CAR-T cell therapy in TNBC, further validation in clinical settings is necessary.

#### c-Met-targeted CAR-T

The hepatocyte growth factor receptor, c-Met, is a cell-surface molecule expressed in 50% of breast cancer tumors rendering it a valuable immunotherapeutic target. In this context, a clinical trial evaluating c-Met as a target antigen was conducted on a small set of six breast cancer patients 38. Each patient was injected with 1ml of mRNA-transfected c-Met-CAR-T cells two days before tumor excision 38. The tumors were observed post-surgery, and found to experience necrosis, hemorrhage, and inflammatory cell infiltration at the injection site. c-Met expression was also lost in post-injection tumors 38. The effectiveness of c-Met as a target antigen for CAR-T cell therapy requires further investigation in larger groups of breast cancer patients.

#### NKG2DL targeted CAR-T

Triple-negative breast cancer cells usually upregulate their expression of stress-induced ligands some of which are recognized by natural-killer group 2, member D (NKG2D) 39,40. These ligands include MICA/B and ULBP 1-5 in breast cancer 39,41. Since the adoptive transfer of NK cells has failed to induce tumor regression, CAR constructs containing full-length NKG2D were tested by Stentman's laboratories 40,42,43. The results of the NKG2D-CAR-T preclinical trial were promising in solid tumors (including breast cancer) and the THINK clinical trial is currently recruiting to test the safety and activity of human NKG2D CAR referred to as NKR-2 43-45.

#### HER2 targeted CAR-T

CAR was transduced in CD3^+^ cells to target HER2 and was found to specifically target and induce apoptosis in the HER2 overexpressing breast cancer cell line 46. Herceptin-based CARs with modified signaling domains generated to target HER2 resulted in antitumor activity in breast cancer cell lines and a breast cancer xenograft mouse model 47. Further studies showed that the adoptive transfer of autologous HER2-specific T-lymphocyte clones to a patient with metastatic HER2-overexpressing breast cancer prevented tumor cell dissemination to the bone marrow. However, the T cells were found unable to penetrate solid tumor metastases masses in the liver 48. Her2-CAR-T therapy requires more optimization as a case study reported multiple organ dysfunction syndrome due to cytokine storm and resulted in the patient's death 49.

#### Experimentally promising CAR-T

##### HERV-K targeted CAR-T

Human endogenous retroviruses (HERVs) account for 8% of the human genome 50. They have been incorporated into the genome millions of years ago after germline infections and are now referred to as “fossil” sequences 50-52. The transcription of HERVs is controlled epigenetically, such that HERV expression is inhibited in normal adult cells 53,54. In diseases where epigenetic mechanisms are disrupted, as in cancer, HERV protein expression is upregulated. Specifically, the expression levels of certain genes in the HERV-K group, such as *env, gag,* and* np9* mRNA were elevated in breast cancer cells and were suggested as biomarkers for early breast cancer diagnosis 55,56. The HERV-K *env* gene is expressed in 70% of breast cancers and is associated with breast cancer progression 57. In addition, the overall survival of breast cancer patients with a high HERV-K env was lower compared to patients with low or moderate expression 57. HERV-K viruses of the HML-2 subtype are the most intact retroviruses in the human genome and can be found in very high titers in the plasma of patients with breast cancer 58,59. The transcripts of the *env* gene of HERV-K HML-2 subtype were shown to induce cancer development, and enhance *in vitro* invasion and migration, and cancer metastasis in MDA-MB 231 xenograft mouse model 58,60. The env protein of HERV-K was found to be an immunogenic tumor-associated antigen and antibodies targeting it possess anti-tumor activity. Zhou et al injected breast cancer patients and normal female donors with anti-HERV-K monoclonal antibodies and derived chimeric antigen receptor (CAR)-T cells specific for HERV-K env protein (K-CAR) from the peripheral blood mononuclear cells of breast cancer patients and normal donors. The single-chain variable fragment was then introduced to the K-CARs and tested both *in vitro* on breast cancer cells and *in vivo* on human tumor xenograft mouse models. A significant decrease in tumor size and weight and cancer cell growth was observed in cells treated with K-CAR compared to control 58,60. Even so, K-CAR may be a promising immunotherapy, more clinical testing is required to validate its safety and efficacy in humans.

##### Folate receptor-alpha targeted CAR-T

Folate receptor-alpha (FR-α) is upregulated in non-mucinous tumors of epithelial origins such as breast cancer; however, its overexpression is mostly detected in the TNBC subtype 61,62. FR-α overexpression was found to be associated with poor outcomes in breast cancer and worse clinical outcomes in TNBC 63,64. Estrogens control FR-α expression in breast cancer, and 17-β-estradiol downregulates its expression by a direct action on the estrogen receptor at the FR-α promoter 62,65. This suggests a negative correlation between ER and FR-α expression, which explains why ER-negative breast cancers express more FR-α 66. FR-α targeted CAR-T showed cytotoxic activity in breast cancer *in vitro* 66,67, and resulted in reduced tumor progression in a TNBC xenograft mouse model 66. More experiments and trials are required and the variability in FR-α expression may demand further patient stratification in clinical studies 68.

##### MUC1 and ErbB2-targeted CAR-T

CAR-T cells engineered to target two antigens MUC1 and ErbB2 have shown successful results in breast cancer *in vitro* 69. Dual targeting is an appealing approach for CAR-T cell therapy because it may reduce the toxic potential of the treatment and promote T-cell survival within the tumor. The reduced toxic potential results from optimized T-cell homing and tumor specificity. Nevertheless, enhanced T-cell survival in the tumor is attributed to the synergistic signals primarily delivered to T cells within the tumor microenvironment 69. *In vivo* studies are still underway to compare the potency, efficacy, and safety of dual targeting.

## Vaccines

### Peptide-based Vaccines

Peptide-based vaccines focus mainly on eliciting a cellular antigen-specific T-cell response against antigens highly expressed on tumor cells such as HER2 and MUC1 in breast cancer 70-72. Since the response to HER2 and MUC1 antibodies is low in breast cancer, peptide vaccines are mostly used with GM-CSF as a cytokine adjuvant to increase efficacy 73.

#### HER2

Vaccines made from peptides that may help the body build an effective immune response against HER2/neu expressing tumor cells are currently in clinical trials. HER2/neu immunogenic peptides vaccines are stretches of peptides from the HER2/neu protein. HER2 immunogenic peptides include G89 (HER-2/neu: 777-789), F7 (HER-2/neu: 776-788), p776 (HER-2/neu: 774-788), AE36 (HER-2/neu:776-790), GP2 (HER2/neu: 654-662), and E75 (HER2/neu: 366-37) 74-77. Fusion proteins with the Ii-key (amino acids 77-80 of the immune-regulatory Ii protein), LRMK sequence, and ε-aminovaleric acid (Ava) were tested for increased antigenicity and T cell response 76. AE37 is a Ii-Key hybrid of AE36 that induces a generalized immune response without the use of an adjuvant 78,79.

The trials that tested AE37, GP2, and E75 with GM-CSF showed that all three peptide vaccines were safe and well-tolerated 80. In phase II clinical trial, the overall intention-to-treat analysis demonstrated no benefit to vaccination. However, the results did confirm the safety of the vaccine and suggested that vaccination may have clinical benefits in patients with low HER2-expressing tumors 81. As for GP2 combination with GM-CSF, the phase II clinical trial did not demonstrate clinical benefit when administered in the adjuvant setting to node-positive and high-risk node-negative breast cancer patients with tumors expressing any degree of HER2 82.

A recently completed clinical trial investigated the safety and clinical efficacy of GP2 and AE37 combined with the immunoadjuvant GM-CSF in high-risk breast cancer patients with any level of HER2 expression in adjuvant settings. Since HLA-A2 status is thought to affect peptide vaccine outcomes, this trial sought to investigate HLA-A2 and HER2 statuses 83. While the results of the HLA-A2 status did not significantly influence outcomes, the HER2 expression in patients highly treated with adjuvant trastuzumab had a significantly better disease-free survival than patients with low HER2 expression 83.

E75 peptide (Nelipepimut-S) was evaluated in phase I/II clinical trial on node-positive and high-risk node-negative breast cancer patients with tumors expressing any degree of HER2 in the adjuvant setting. The five-year disease-free survival rate was 94.6% in optimally dosed patients (P = 0.05 versus the CG) and 87.1% in suboptimally dosed patients 84. A phase III clinical trial tested the efficacy of Nelipepimut-S with GM-CSF in preventing breast cancer metastasis. The results showed that the treatment could not significantly decrease recurrence in HER2 low node-positive breast cancer patients in the adjuvant settings 85. Synergistic effects have been observed when passive immunotherapy (monoclonal antibodies) is combined with active immunotherapy (cancer vaccines). Preclinical studies in murine models have shown that both cellular and humoral anti-neu immune responses are necessary to eliminate HER2/neu-expressing tumors 86-89. This mechanism occurs through antibody-dependent cellular cytotoxicity mediated by natural killer cells, such that the antibody-induced cytotoxicity causes the tumor cells to lyse and release antibody-coated tumor antigens 90-92. Further studies are required on HER2/neu vaccines to know their efficacy compared to trastuzumab.

#### MUC1

Due to their low antibody immune response, MUC1 peptide vaccines have been used in various combinatorial approaches. One such promising combination uses Bacille Calmette-Guérin (BCG) conjugated with the MUC1 antigen and human interleukin-2 (IL-2) vaccine. The preclinical study showed an increased CD8^+^ T cell response and inhibited breast cancer growth 73,93. Further clinical trials are required to test its safety and efficacy in humans. Another trial targeted MUC1 using liposomal BLP25 (L-BLP25; a homolog of the protein backbone of MUC1) vaccination therapy, Tecemotide. The results of this study have been recently published and showed no significant increase in the residual cancer burden or pathologic complete response between patients taking Tecemotide and the control group 94.

### Gene-based vaccine

Gene-based vaccines can be either (i) recombinant viral vectors modified to express tumor-associated antigens or (ii) bacterial plasmids constructed to function as a shuttle system that delivers and expresses a tumor antigen to help activate targeted cellular and humoral immunity 93,95.

#### Viral vaccines

Viral vaccines have been tested in breast cancer, such as the Modified Vaccinia Ankara (MVA) which consists of the Twist transgene and a TRIad of COstimulatory Molecules (B7-1, ICAM-1, LFA-3; TRICOM) (MVA-TWIST/TRICOM). This vaccine was shown to induce both CD8^+^ and CD4^+^ T cell responses against the transcription factor Twist, thereby reducing tumor growth and metastasis in a metastatic breast cancer model 96. Viral vaccines, however, pose a clinical risk as they can induce strong immunity to the viral constructs and the production of neutralizing antibodies 97.

#### DNA vaccines

In breast cancer, DNA vaccines are combined with immunostimulatory molecules, such as toll-like receptors (TLR). The combination of DNA vaccines targeting HER2 with the agonist TLR9 showed potent anti-tumor activity and antibody-dependent cytotoxicity in mice 98. Another DNA vaccine was designed by fusing the extracellular domain of CTLA-4 to HER-2/Neu to facilitate the detection of tumor antigens by APCs. This DNA vaccine-induced protective humoral and cellular immune responses, which delayed the onset of spontaneous Neu-driven mammary carcinomas and improved tumor-free survival of HER-2/Neu-driven mammary carcinoma in mice 99. Similar to viral vaccines, DNA vaccines are not translated successfully in clinical settings due to efficacy rather than toxicity, and thus more improvements on the constructs and methods of administration are under investigation to enhance their utility in patients.

### Whole-cell vaccine

Instead of using a specific antigen, whole tumor lysates were investigated for their immunogenicity. Whole-cell tumor vaccines, prepared by irradiating autogenic or allogenic cancer cells, increased cancer recognition by CTL and antigen-presenting cells 100. Whole cell-based vaccines may thus induce a broader immune response given the broader load of antigenic components with a high yet rare risk of developing autoimmune diseases 101,102.

However, current research has shown that they are poorly immunogenic 103. Modifications to these vaccines have been evaluated, such as modifying the irradiated cancer cells to secrete GM-CSF, in an attempt to have a better effect when combined with chemotherapy 104. Other modifications included the addition of IL2, VEGFR2, and BCG in adjuvant settings to improve the immunogenicity and efficacy of whole-cell vaccines 105,106. The whole-cell vaccine with BCG adjuvant showed improved survival in 60% of breast cancer patients after a 5-year follow-up 106. The vaccination of a murine mammary cancer model with whole-cancer cells infected with irradiated adenovirus encoding VEGFR2 inhibited subsequent tumor growth, angiogenesis, and pulmonary metastasis. In addition, the number of CD8^+^ T lymphocytes was increased within the tumors of vaccinated mice 107. This modified whole-cell lysate vaccine may be a potentially effective strategy for breast cancer treatment.

### Dendritic cell-based vaccine

Dendritic cells (DC) are antigen-presenting cells that help in activating the anti-tumor immune response. DCs recognize the antigen on tumor cells, phagocytose and process the antigen, and present it on their cell surface to prime and activate cytotoxic T lymphocytes (CTL) 108. DCs can be activated against tumor antigens *in vitro,* modified genetically using recombinant viral vectors, or fused with tumor cells using polyethylene glycol (PEG) or electrofusion to maintain antigen presence 109,110.

Lapuleucel-T vaccine is an example of *in vitro* activated DCs. It is derived from autologous peripheral blood mononuclear cells, including antigen-presenting cells overexpressing HER-2/neu-GM-CSF. This vaccine was tested in HER2^+^ metastatic patients and resulted in induced T cell response and tolerability 111.

Genetically modified DCs generated via recombinant adenoviral transduction of bone marrow-derived DCs to express a truncated HER2 protein were developed and tested *in vivo.* The vaccine increased the production of anti-HER2 antibodies, enhanced T cell response, and delayed the onset of mammary carcinomas in mice 110.

The fusion of DCs with tumor cells was tested *in vitro* and *in vivo.* This DC vaccine showed a high CTL response and resulted in the eradication of the tumor within 90 days 112. However, these results were not further corroborated as other studies on PEG fusion-based DC vaccines were conducted on a mammary dog model using autologous and allogenic cancer cells and did not show an effective response as in the mouse model 113,114.

A more recent study sought to test the anti-tumor ability of DC administration in combination with chemotherapy in a mouse breast cancer model 115. After adjusting the dosage of paclitaxel to minimize T cell inhibition, the combined treatment of paclitaxel and DC was found to induce antigen-specific CD8-mediated response in all 9 tested mice and CD4-mediated response in 6 out of the 9 treated mice. Furthermore, the group of mice treated with DC and paclitaxel had a significantly longer survival (45 days) compared to the untreated control group (29 days) and the tumor size was diminished compared to paclitaxel or DC administration alone. Therefore, the combination of paclitaxel and dendritic cells may be a potentially successful treatment in breast cancer 115.

Another clinical trial examined the effect of DC in combination with adoptive cell transfer, cytokine-induced killer T-cell therapy, and high dose chemotherapy on 166 metastatic breast cancer patients. This combination aims to eliminate the chemotherapeutic-resistant cancer stem cells to improve response to chemotherapy. The control group received a standard dose of chemotherapy (75 mg/m^2^ docetaxel and 75 mg/m^2^ thiotepa); while the treatment group received high-dose chemotherapy (120 mg/m^2^) in combination with DC and autologous cytokine-induced killer immunotherapy (DC/CIK) 116. The results of the study showed that the treatment group had a significantly improved progression-free survival and overall survival compared to the control group 116. Another study investigated the role of DC vaccines in targeting breast cancer stem cells *in vitro* and* in vivo.* DCs loaded with 4T1 tumor antigens improved CTL response against breast cancer stem cells *in vitro* and decreased tumor size *in vivo* 117*.* More clinical trials including combination therapies with DC are listed in Table 1.

### Checkpoint inhibitors

#### Anti-PD1/PDL1 inhibitors

Expressed on T-cells, B cells, and NK cells, programmed cell death 1 (PD-1) is a checkpoint that keeps the immune response in check to prevent autoimmune diseases and immune overactivation 118,119. The inhibition of PD-1 occurs when it binds to its ligands, PD-L1 and PD-L2, which are commonly expressed on myeloid and tumor cells 119-121. A meta-analysis of five studies (2,546 patients) has shown that PD-L1 positive expression in breast cancer ranges between 21.7% to 56.6% 122. The expression levels of PD-1 and its ligand, PD-L1, vary depending on the subtype of breast cancer. For example, basal-like breast cancer has the highest overall PD-1 expression on Tumor-Infiltrating Lymphocytes (TILs) (27.4%) while the luminal A subtype has the lowest overall expression (4.7%). Furthermore, PD-L1 expression has been associated with positive lymph node metastasis, higher histological grade, larger tumor size, triple-negative subtype, and HER2 positivity 123.

Drugs targeting the PD-1/PD-L1/PD-L2 signaling pathway block the interaction between PD-1 and its ligands by targeting either PD-1 (Pembrolizumab and Nivolumab) or PD-L1 (Atezolizumab, Avelumab, and Durvalumab) (Figure 2B). While it is thought that patients with a high PD-1 expression would respond well to anti-PD-1 treatments, the response is highly variable among patients. This is mainly due to the lack of well-defined cutoff criteria, technical discrepancies, and T cell infiltration to the tumor 124,125. Furthermore, a variety of genetic aberrations can cause a constitutive PD-L1 expression, which may arbitrarily induce PD-L1 positivity regardless of the presence or absence of T-cell infiltration. Recently, we have identified a list of biomarkers that classify the subsets of breast cancer patients that would respond best to checkpoint inhibitors or other combinations of immunotherapies 126.

Several studies have shown that PD-1/PD-L1 signaling antagonists induce clinical response durability in some metastatic TNBC patients. This has led to the FDA approval of the first immunotherapy in breast cancer, anti-PD-L1 (atezolizumab) in combination with chemotherapy (nab-paclitaxel) for metastatic TNBC 127,128. The phase III trial showed prolonged progression-free survival from 5.5 months in the placebo group plus nab-paclitaxel to about 7.2 months for the atezolizumab plus nab-paclitaxel combination group. The PD-L1 positive subgroup also had a prolonged progression-free survival of 7.5 months in the combination group compared to 5 months in the control group 127. However, several questions have been raised including how to enrich the responsive TNBC population, how to assess PD-L1 positivity and thus patients' response to atezolizumab, whether PD-L1 expression should be tested in the tumor or immune cells, if nab-paclitaxel is the best chemotherapy to use and if atezolizumab monotherapy could have been beneficial for a certain subset of patients without chemotherapy 129. Several studies have shown the relevance of these questions. We have classified the TNBC patients into 2 clusters, one that would benefit from chemotherapy and the other would benefit from anti-PD-L1 treatment in combination with other immunotherapeutic drugs, such as anti-CTLA4 126,130. A concise review on how to enrich the TNBC population is addressed by Marra *et al.* 129.

Multiple completed trials have tested the effects of PD-1 inhibitors as monotherapies in breast cancer patients. The Keynote-086 phase II trial evaluated pembrolizumab as first- or later-line treatment for PDL-1 positive patients with metastatic TNBC untreated or previously treated with chemotherapy 131,132. In the previously treated cohort, the overall response rate (5.3%) was lower than single-agent chemotherapy. However, pembrolizumab abolished the common toxicities associated with chemotherapy and resulted in durable responses. In addition, 75.0% and 62.5% of responders had a response duration of ≥6 and ≥12 months, respectively compared to the typical duration of response (1-3 months) seen in standard chemotherapy in the metastatic TNBC setting. Thus, pembrolizumab showed a durable effect in patients who achieved a response 132. In the previously untreated cohort, pembrolizumab monotherapy demonstrated an acceptable safety profile despite the common adverse effects occurring in 63% of patients. The antitumor activity was robust and durable with a median time to response of 2.0 months, a median duration of response of 10.4 months, median overall survival of 18 months, and an overall survival rate of 48.7% at 18 months. Thus, pembrolizumab demonstrated antitumor durability as a first line of treatment.

One combinatorial approach involved the blockade of PD-1/PD-L1 signaling together with CTLA4. A pilot study evaluated durvalumab (anti-PD-1 drug) and tremelimumab (CTLA4 blocking drug) in refractory metastatic breast cancer patients 133. The overall response rate was 17% which was mostly seen in TNBC patients (43%) with no response to treatment detected in the ER-positive patients. This study also found that TNBC patients who did respond to treatments had a higher non-synonymous mutation load along with a higher prevalence of neoantigens; both of which increased the number of activated T cells 133-135. Despite the limitations attributed to this study, it provides important observations to plan future trials. Other ongoing clinical trials are summarized in Table 1.

#### Anti-CTLA-4 inhibitors

Cytotoxic T lymphocyte antigen 4 (CTLA4) is an immune checkpoint molecule typically expressed on the surface of regulatory T-cells and cytotoxic T cells shortly after activation 121,136. CTLA4 limits T-cell activation by interacting with its two ligands CD80 and CD86 (Figure 2C) 137. A recent study found that high CTLA4 expression is associated with poor prognosis in breast cancer 136.

Only a few studies investigated the effects of CTLA4 blockade on breast cancer, most of which combined CTLA4 blocking drugs with other treatments such as aromatase inhibitors, radiotherapy, and chemotherapy.

##### Anti-CTLA4 + aromatase inhibitor

The effect of tremelimumab (anti-CTLA-4 antibody) in combination with exemestane - a steroidal aromatase inhibitor - was evaluated in breast cancer 2. The study was done on postmenopausal women with life expectancies greater than six months who had estrogen receptor-positive breast cancer except for one 137. Forty-two percent of the patients developed a stable disease; however, none of the patients experienced partial or objective responses. It should be noted that 36% of the patients who developed a stable disease had previously experienced tumor progression while on exemestane 137.

##### Anti-CTLA4 + radiotherapy

Anti-CTLA4 was tested in combination with single-dose and fractionated radiotherapy in a mouse breast cancer model to determine if it can produce what is known as an “abscopal effect” on secondary and primary tumors. The abscopal effect is tumor regression that occurs outside of the field of radiation 138. It was found that CTLA4 blockade had no effect on its own but caused tumor regression when combined with radiotherapy. However, the abscopal effect to the secondary tumor only occurred when radiotherapy doses were fragmented and not given in single-dose form 138. Furthermore, the higher radiation regimen (8Gy) showed a more efficient abscopal effect than a lower dose (6Gy) regimen 138.

##### Anti-CTLA4 + chemotherapy

Anti-CTLA-4 combination with metronomic chemotherapy has also been tested in breast cancer 139. Metronomic chemotherapy is a type of chemotherapy that involves the administration of a chemotherapeutic drug in relatively low doses over a long period to prevent the tumor from becoming drug-resistant 140. This combination was investigated in preclinical settings in breast cancer and metronomic cyclophosphamide was shown to enhance the outcome of anti-CTLA4 therapy. CTLA4 antibody administration alone decreased tumor size in mice but resulted in tumor relapse 139. More impressive results were seen using a sequential regimen of CTLA4 blockade followed by metronomic gemcitabine chemotherapy 139,141. Despite these results, resistance was still observed as well as spontaneous metastases.

## Conclusion

Several advances have been made to increase breast cancer patient's survival and mitigate tumor growth. Approvals have been granted for several targeted therapies and combination therapies and 2019 highlights the first approved immunotherapy in combination with chemotherapy for metastatic TNBC. More improvements, however, can be made by taking a more personalized approach to tackle the intra- and inter-tumor heterogeneities, and by identifying better biomarkers to make the personalization more clinically feasible. Thus, more basic, pre-clinical, translational, and clinical investigations are required to enhance patients' response by further categorizing patients based on their tumor phenotypic and genotypic characteristics.

## Supplementary Material

Supplementary data.

## Figures and Tables

**Table 1:** Current breast cancer immunotherapy trials

Therapy	Condition	Trial	Trial ID	Phase
DC	Stage II and III	Chemotherapy followed by Autologous DC and surgery with/without radiation and/or hormone Therapy	NCT00499083	II
	Locally recurrent or metastatic	Vaccine therapy, trastuzumab, and vinorelbine	NCT00266110	II
	TNBC and ER^+^/HER2^-^	Safety trial for chemotherapy and DC vaccine	NCT02018458	I, II
	P53 overexpression and Stage III	Vaccine therapy + adjuvant/neoadjuvant chemotherapy + adjuvant radiation therapy	NCT00082641	I, II
	Metastatic BC	Vaccine therapy + 1-MT	NCT01042535	I, II
	Stage IV	DC/tumor fusion + IL12	NCT00622401	I, II
	HER-2 driven invasive breast cancer at least Stage IIIA	HER-2 pulsed Dendritic Cell Vaccine	NCT02063724	I
	Locally recurrent/metastatic BC	Vaccine therapy + with trastuzumab + vinorelbine	NCT00088985	II
Adoptive T-cell therapy	Stage IV	*Ex vivo*-expanded HER2-specific T cells	NCT00791037	I, II
Anti-PD-1/PD-L1	Advanced, trastuzumab-resistant, HER2^+^ BC	Ant-PD-1 (Pembrolizumab; MK-3475)	NCT02129556	I, II
	Metastatic TNBC	Pembrolizumab + radiotherapy	NCT02730130	II
	TNBC	Pembrolizumab (MK-3475) + chemotherapy as neoadjuvant treatment	NCT02622074	I
	Advanced/metastatic TNBC and OC	Niraparib + pembrolizumab	NCT02657889	I, II
	HER2 overexpressing metastatic BC	Pembrolizumab + monoclonal antibody therapy	NCT02318901	
	TNBC	Pembrolizumab + epacadostat	NCT02178722	I, II
		Trastuzumab emtansine + atezolizumab/atezolizumab-placebo	NCT02924883	II
	Metastatic TNBC	Cobimetinib (MEK inhibitor) + paclitaxel, Cobimetinib + atezolizumab Plus Paclitaxel, or Cobimetinib + atezolizumab + Nab-Paclitaxel	NCT02322814	II
	Relapsed/refractory BC	Ibrutinib + durvalumab (MEDI4736)	NCT02403271	I, II
	Stage IV HER2^-^ BC	Ipilimumab and Nivolumab	NCT02892734	II
	Metastatic TNBC	SYK inhibitor (TAK-659) + nivolumab	NCT02834247	I
	TNBC	Anti-PDL1 (MEDI4736) monotherapy or MEDI4736 + tremelimumab (anti-CTLA4)	NCT02527434	II
Anti-CTLA4	Recurrent Stage IV HER2^-^ BC	Ipilimumab and Nivolumab	NCT02892734	II
				

BC: Breast cancer; 1MT: 1-methyl-d-tryptophan; OC: Ovarian cancer; TNBC: triple-negative breast cancer; HER2: Human epidermal growth factor receptor 2; ER: estrogen receptor, IL: interleukin.

## Abbreviations

APC: antigen-presenting cell
BCG: Bacille Calmette-Guérin
BRCA: Breast cancer
CAR: Chimeric antigen receptor
CD: Cluster of differentiation
CDK: Cyclin-dependent kinase
CIK: Cytokine-induced killer
c-Met: tyrosine-protein kinase Met or hepatocyte growth factor receptor (HGFR)
CTL: Cytotoxic T-lymphocyte
CTLA4: Cytotoxic T-lymphocyte-associated protein 4
DC: Dendritic cell
DSB: Double-stranded break
E2F: E2 transcription factor
EGFR: Epidermal Growth Factor Receptor
ER: Estrogen receptor
ErbB2: Human epidermal growth factor receptor 2 (HER2)
FDA: Food and drug administration
FRα: Folate receptor-alpha
GM-CSF: Granulocyte monocyte colony-stimulating factor
HER2: Human epidermal growth factor receptor 2
HERV-K: Human endogenous retrovirus-K
HLA: Human leukocyte antigen
HML-2: Human endogenous retrovirus-K
HR: Hormone receptor
IL2: Interleukin 2
MUC1: Mucin-1
MVA: Modified vaccinia Ankara
Nab: Nanoparticle albumin-bound
NKG2D: Natural killer group 2 member D
PARP: Poly(ADP-ribose) polymerase
PD1: Programmed death 1
PDL1: Programmed death-ligand 1
PEG: Polyethylene glycol
PR: Progesterone receptor
RB: Retinoblastoma
ScFV: Single-chain variable fragment
SSB: Single-stranded break
TCR: T cell receptor
TLR: Toll-like receptor
TNBC: Triple-negative breast cancer
VEGFR2: Vascular endothelial growth factor receptor 2
